# A *TSC2* recurrent variant c.5126C>T in a Han-Chinese family with tuberous sclerosis complex

**DOI:** 10.12669/pjms.41.1.10153

**Published:** 2025-01

**Authors:** Xinyue Deng, Shan Wu, Hao Deng, Lamei Yuan

**Affiliations:** 1 Xinyue Deng, Health Management Center, the Third Xiangya Hospital, Disease Genome Research Center, Center for Experimental Medicine, the Third Xiangya Hospital, Research Center of Medical Experimental Technology, the Third Xiangya Hospital, Xiangya School of Medicine, Central South University, Changsha 410013, Hunan, China; 2 Shan Wu, MS, Health Management Center, the Third Xiangya Hospital, Disease Genome Research Center, Center for Experimental Medicine, the Third Xiangya Hospital, Research Center of Medical Experimental Technology, the Third Xiangya Hospital, Xiangya School of Medicine, Central South University, Changsha 410013, Hunan, China; 3 Hao Deng, MD, PhD, Health Management Center, the Third Xiangya Hospital, Disease Genome Research Center, Center for Experimental Medicine, the Third Xiangya Hospital, Research Center of Medical Experimental Technology, the Third Xiangya Hospital, Xiangya School of Medicine, Central South University, Changsha 410013, Hunan, China; 4 Lamei Yuan, MD, PhD, Health Management Center, the Third Xiangya Hospital, Disease Genome Research Center, Center for Experimental Medicine, the Third Xiangya Hospital, Research Center of Medical Experimental Technology, the Third Xiangya Hospital, Xiangya School of Medicine, Central South University, Changsha 410013, Hunan, China

**Keywords:** Tuberous sclerosis complex, The *TSC2* gene, Whole exome sequencing, Recurrent variant, Epilepsy

## Abstract

**Objective:**

To identify the disease-causing variant in a family with tuberous sclerosis complex (TSC).

**Methods:**

This study including a Han-Chinese pedigree recruited from the Third Xiangya Hospital, Central South University, Changsha, Hunan, China was conducted between February, 2019 and January, 2023. Detailed clinical examinations were performed on the proband and other family members of a Han-Chinese family with TSC. Whole exome sequencing of the proband and Sanger sequencing of all family members were performed, followed by variant pathogenicity prediction and conservation analysis. SWISS-MODEL and PyMOL software were used for protein modelling and creating the three-dimensional structure model illustration of the critical GTPase-activating protein (GAP) domain. The variant was classified following the American College of Medical Genetics and Genomics (ACMG) standards and guidelines.

**Results:**

The female proband exhibited typical features of TSC, including hypomelanotic macules, angiofibromas, shagreen patches, seizures, brain lesions, cognitive impairment, renal abnormalities, and cardiovascular abnormalities. A recurrent c.5126C>T variant in the TSC complex subunit 2 gene (*TSC2*) was identified as the genetic cause of TSC in this family, classified as “pathogenic” according to ACMG standards and guidelines. The c.5126C>T variant leads to an amino acid change from proline to leucine at position 1709 (p.P1709L) in the functional GAP domain of tuberin protein, which may impair tumor growth inhibition of the hamartin-tuberin complex.

**Conclusion:**

This study reported a Han-Chinese TSC patient with a recurrent variant *TSC2* c.5126C>T (p.P1709L). These findings broaden the phenotypic spectrum of TSC caused by this variant and may contribute to improving TSC genetic diagnoses as well as understanding of its mechanisms.

## INTRODUCTION

Tuberous sclerosis complex (TSC) is a rare multisystem genetic disorder with an estimated birth incidence of 1:6000-1:10,000.^1^ The clinical manifestations of TSC are highly variable, characterized by benign tumors in diverse organ systems, including the brain, kidney, lung, skin, heart, and eyes.^2^ Most TSC patients come to medical attention due to epilepsy or dermatologic features, while the primary causes of death among these patients are neurological causes, renal complications, and lymphangioleiomyomatosis.^3^ TSC is an autosomal dominant disease, though approximately two-thirds of cases carrying *de novo* pathogenic variants were reported as sporadic.^4^ Two tumor suppressor genes are responsible for TSC, the TSC complex subunit 1 gene (*TSC1*) and the TSC complex subunit 2 gene (*TSC2*).

*TSC1* and *TSC2* encode the 130-kDa protein “hamartin” and the 200-kDa protein “tuberin” respectively.^5^ Hamartin and tuberin can form a complex which plays a role in inhibiting of the mammalian target of rapamycin (mTOR), which regulates the signalling pathway of cell growth and proliferation.^6^ As of 2024, at least 648 *TSC1* variants and more than 1970 *TSC2* variants have been recorded in the Human Gene Mutation Database (HGMD Professional 2023.4, accessed October 2nd, 2024). Identification of a pathogenic variant or the deletion in either *TSC1* or *TSC2* is regarded to be adequate for confirming a TSC diagnosis, independent of clinical observations. This study identified a recurrent heterozygous variant c.5126C>T (p.P1709L) in *TSC2* through whole exome sequencing combined with Sanger sequencing, which was considered as the genetic cause in a TSC-afflicted Chinese family.

## METHODS

A Han-Chinese pedigree including four individuals was recruited from the Third Xiangya Hospital, Central South University, Changsha, Hunan, China. This study was conducted between February, 2019 and January, 2023.

### Ethical Approval:

This study was performed according to the Declaration of Helsinki and gained Ethics Committee approval from the Institutional Review Board of the Third Xiangya Hospital, Central South University, Changsha, Hunan, China (No: 2018-S400, dated December 26, 2018).

After written informed consent was obtained from each participant or their legal guardian, the proband of the Han-Chinese family, her elder brother, and her parents were enrolled in the study. Medical history revealed that her parents and her brother were normal. Detailed physical examinations, video electroencephalography, brain magnetic resonance imaging (MRI), electrocardiography, abdominal ultrasound, and neuropsychological evaluations including the Mini-Mental State Examination (MMSE) and the Montreal Cognitive Assessment (MoCA) were performed on the proband and other family members. TSC diagnosis was established according to the latest diagnostic criteria recommended by the 2021 International Tuberous Sclerosis Complex Consensus Group.^1^ Peripheral blood samples were collected from the proband (II:2) and additional three family members (I:1, I:2, and II:1, Fig.1A).

**Fig.1:**
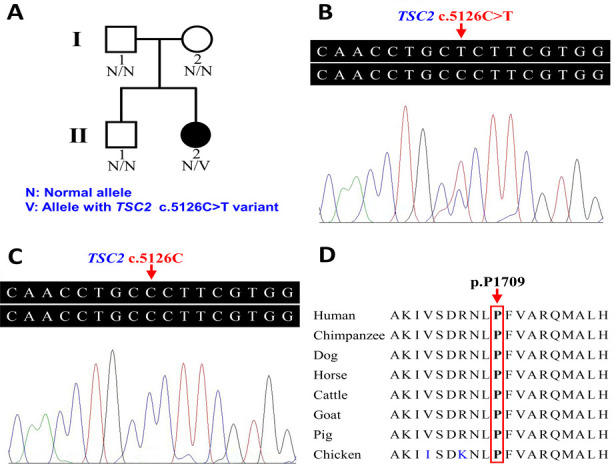
Pedigree and sequence analysis of a Han-Chinese family with TSC. (A) Pedigree of the family with TSC. (B) DNA sequencing of a heterozygous *TSC2* c.5126C>T variant in the proband (II:2). (C) DNA sequencing of wild-type *TSC2* gene in unaffected member (II:1). (D) Conservation anal-ysis of TSC2 p.P1709 amino acid residue.

### Genetic Analysis:

Genomic DNA was extracted as previously described, and 1 μg genomic DNA of the proband was broken into fragments by Covaris method. DNA fragments between 150-250 bp were selected and end-repaired further. These fragments were then amplified, purified, and hybridized for enrichment. The qualified captured library was sequenced on the BGISEQ-500 sequencing platform. Clean data was generated by filtering raw data. Burrows-Wheeler Aligner software (v0.7.15) was used to align the clean data to the human reference genome.

The Genome Analysis Toolkit (GATK, v3.3) was used for local realignment and base quality score recalibration. Picard tools (v2.5.0) was utilized to remove duplicate reads. The HaplotypeCaller of GATK was used to call variants including single nucleotide polymorphisms (SNPs) and insertions and deletions (InDels). The SnpEff tool was used for high-confident SNPs and InDels annotation. All variants were filtered against public databases such as the Single Nucleotide Polymorphism database (dbSNP, version 154) and the 1000 Genomes Project database. A total of 3313 controls, including 842 Han-Chinese controls without any TSC diagnostic features from our in-house exome database and the BGI in-house exome database containing 2471 Chinese controls were used to further filter the remaining variants.^7^

The pathogenicity of variants was predicted by *in silico* tools, including MutationTaster, Polymorphism Phenotyping version 2 (PolyPhen-2), MutationAssessor, Functional Analysis Through Hidden Markov Models (FATHMM), and Combined Annotation Dependent Depletion (CADD). Candidate variant was verified in all family members via Sanger sequencing on an ABI3500 sequencer (Thermo Fisher Scientific, Waltham, MA, USA). Locus-specific primers were designed using the Primer3 (http://primer3.ut.ee/). The sequences of primers using for the causal variant detection were: 5’-CACCAAGTCTCCCCAGACA-3’ and 5’-TAGATATCGGTGGGGTTGGA-3’. Conservation analysis was performed by Basic Local Alignment Search Tool (BLAST) in National Center for Biotechnology Information (NCBI). Protein modelling was done for wild-type GTPase-activating protein (GAP) domain and p.P1709L mutant type GAP domain of TSC2 by SWISS-MODEL (http://swissmodel.expasy.org). Using PyMOL software, the three-dimensional structure model illustration was created. Computed structure model of Rap-GAP domain-containing protein (A0A4W5KLM2) from AlphaFold database was used as the template for modelling.^8^ According to the American College of Medical Genetics and Genomics (ACMG) standards and guidelines, the candidate variant was evaluated and classified.^9^

## RESULTS

**Fig.2:**
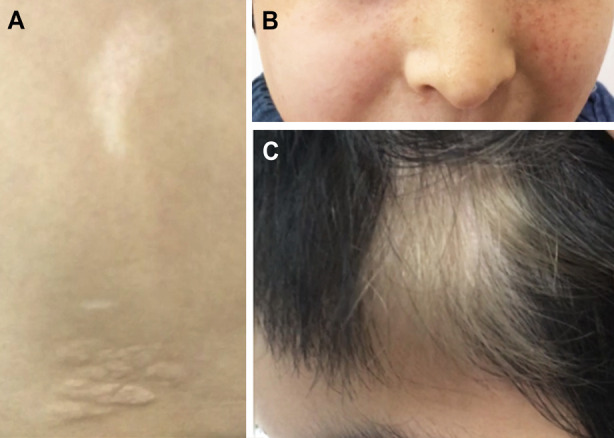
The proband’s dermatologic manifestations. (A) Hypomelanotic macules and shagreen patches. (B) Facial angiofibroma. (C) A circumscribed area of hypomelanosis of hair.

The female proband (II:2, Fig.1A) was 17 years old. Seizures were first observed at 11 months. Physical examination showed dermatologic features, including hypomelanotic macules on the back and bilateral thighs, angiofibromas distributed on the face, and shagreen patches on the back (Fig.2A, 2B). In addition, a circumscribed area of hypomelanosis within the proband’s hair was detected and considered as a clinical feature in the count of hypomelanotic macules (Fig.2C). Cerebral MRI and abdominal ultrasound revealed subcortical tubers and left renal cysts, respectively. An electrocardiogram showed that the proband had cardiac arrhythmia. Her MMSE and MoCA score was 10/30 and 9/30, respectively, which suggested cognitive impairment. The proband’s detailed clinical presentations are presented in Table-I. No diagnostic features of TSC were evident in other familial members. A total of 80.91 million effective reads were generated via whole exome sequencing of the proband. The average sequencing depth on target regions was 92.32, and 98.77% of targeted bases were covered by at least 10× coverage. A total of 92,432 SNPs and 14,207 InDels were obtained. Commonly known variants reported in either the dbSNP or the 1000 Genomes Project with a minor allele frequency ≥0.01, as well as synonymous variants, were removed. A heterozygous missense variant, c.5126C>T (p.P1709L) in *TSC2* (NM_000548.5), was considered as a potential disease-causing variant by MutationTaster, PolyPhen-2, MutationAssessor, FATHMM, and CADD. No other potential TSC-related disease-causing variants were found. Sanger sequencing confirmed the *TSC2* c.5126C>T variant in the proband (Fig.1B).

**Table-I:** Clinical presentations of the patients with the c.5126C>T (p.P1709L) variant of *TSC2.*

subject	Patient 1	Patient 2	Patient 3	Patient 4	Patient 5	Patient 6	Patient 7	Patient 8	Patient 9
Reference	This study	Wilson et al. (1996)^11^	Franz et al. (2001)^12^	van Eeghen et al. (2013)^13^	Cai et al. (2017)^14^	Ogórek et al. (2020)^15^	Ogórek et al. (2020)^15^	Chuan et al. (2022)^16^	Togi et al. (2022)^17^
Sex	Female	/	Female	/	/	Female	Female	Male	Male
Age at examination (yr)	16	/	37	/	/	2	2	1	1
Hypomelanotic macules	+	+	/	/	/	+	+	+	/
Angiofibromas	+	+	+	/	/	+	−	/	−
Ungual fibromas	−	+	/	/	/	−	−	/	−
Shagreen patches	+	−	/	/	/	−	−	/	−
Seizures	+	+	/	+	/	+	+	+	−
Brain lesions	+	+	/	/	/	+	+	+	+
Cognitive impairment	+	+	/	/	/	+	+	/	−
Renal abnormalities	+	+	+	/	+	+	+	/	/
Pulmonary abnormalities	−	/	+	/	/	/	/	+	/
Dental features	−	/	/	/	/	−	−	/	/
Cardiovascular abnormalities	+	/	/	/	/	+	+	/	/
Ophthalmologic abnormalities	−	+	/	/	/	−	−	/	−
Inheritance	*De novo*	Sporadic *	Sporadic	/	Sporadic	Sporadic	Sporadic	/	Sporadic

yr: year(s); /: not available; +: present; −: absent.

* The c.5126C>T (p.P1709L) variant reported in patient 2 was described as 5075C>T (Pro1186Leu) in the literature.^11^

**Fig.3:**
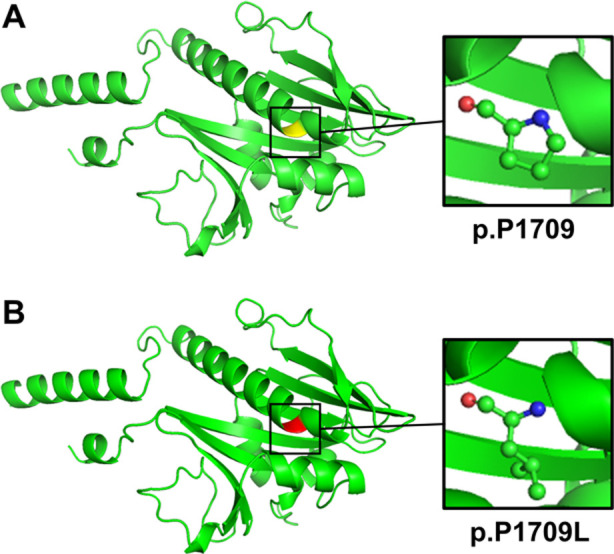
Cartoon model of TSC2 GTPase-activating protein (GAP) domain structure by PyMOL based on SWISS-MODEL. (A) The wild-type proline and (B) the mutated leucine at position 1709 are marked and further shown as ball-and-stick models.

This variant was identified in the proband but was absent from her unaffected parents and brother (Fig.1C). It was also absent in 3313 controls from in-house exome databases. Conservation analysis by NCBI BLAST revealed proline residue at position 1709 was significantly conserved, suggesting an important role in the tuberin structure and function (Fig.1D). Structural analyses of the variant showed it may lead to an amino acid change from proline to leucine in a long helical region of the GAP domain (Fig.3). According to the ACMG standards and guidelines for the interpretation of sequence variants, the identified variant, *TSC2* c.5126C>T (p.P1709L), was classified as “pathogenic”.

## DISCUSSION

In this study, a heterozygous *TSC2* variant c.5126C>T (p.P1709L) was identified in a Han-Chinese family afflicted by TSC. The variant identified in the proband was absent from her parents and brother, suggesting that it was a *de novo* variant. The 17-year-old patient showed dermatologic features, including hypomelanotic macules, angiofibromas, and shagreen patches. She also exhibited seizures and cognitive impairment. In addition, abnormalities in the brain, kidney, and heart were detected. The recurrent *TSC2* c.5126C>T variant was previously noted in distinguishable ethnic origins, indicating that it may be a hot variant spot rather than a founder variant.^10^-^17^ Most patients with this variant were sporadic and presented with similar clinical manifestations, including dermatologic features, epilepsy, brain lesions, and renal abnormalities, while detailed clinical descriptions for some patients with *TSC2* c.5126C>T variant were absent in previous reports (Table-I).

TSC is a multisystem genetic disease with loss-of-function variants in two causative genes, *TSC1* and *TSC2*. As diverse *TSC1* and *TSC2* variants result in a range of clinical manifestations, investigating the correlations between phenotype and genotype in individuals with TSC poses a challenge.^2^ For the *TSC2* c.5126C>T variant, more comprehensive clinical examinations were performed on our reported Han-Chinese patient, revealing clinical features such as shagreen patches, which were not documented in previous patients with the same variant. These findings broaden the phenotypic spectrum of TSC, contribute to the growing evidence regarding the genetic basis of TSC across different ethnicities, and may be helpful to understand TSC genotype-phenotype correlations.

The *TSC2* gene is mapped to chromosome 16p13.3 and contains 42 exons.^4^ The protein product encoded by the *TSC2* gene, known as tuberin, could form a functional complex containing hamartin, tuberin, and Tre2-Bub2-Cdc16-1 domain family member 7, which serves as a critical inhibitor of tumor growth. Its N-terminal region interacts with hamartin, and its C-terminal GAP domain regulates the activity of the GTPase, Ras homolog enriched in brain (Rheb).^18^,^19^ Rheb could stimulate the mTOR pathway, which plays an important role in cell growth, autophagy, and metabolic activity.^6^,^20^ Pathogenic variants of the *TSC2* gene impair the normal function of hamartin-tuberin complex and permanently activate the mTOR pathway, resulting in uncontrolled cellular activities including growth, proliferation, adhesion, migration, and apoptosis.^3^,^4^ This may be the molecular basis of tumors observed in TSC patients. TSC-associated missense variants were more common in *TSC2* than in *TSC1*, and particularly occurred in the GAP domain, indicating its pivotal role in the function of hamartin-tuberin complex.^21^ Several animal models, including conventional and conditional *Tsc2* knockout mice, showed epilepsy, brain anomalies, and tumors in kidney, spleen, and uterus.^22^,^23^ Mice heterozygous for loss of *Tsc2* exhibited more severe TSC-associated features and occurred earlier than those with heterozygous loss of *Tsc1*.^5^,^24^

The p.P1709L variant is located at the GAP domain, a functionally conserved domain including amino acids 1531 to 1758 (UniProt accession: P49815). The proline residue containing an imino group was thought to provide rigidity to the protein structure, which may contribute to the proper arrangement and functional integrity of the GAP domain. The substituted leucine residue lacks the required rigidity and may adversely impact normal cell division inhibition which relies on the indirect regulation of the GAP domain to mTOR.^25^ Previous functional research reported that the p.P1709L variant increased the mTOR pathway activity.^10^ Integrating all the evidence, the *TSC2* c.5126C>T (p.P1709L) variant was classified as a pathogenic variant following the ACMG guidelines. Advances in next-generation sequencing, such as whole genome sequencing and whole exome sequencing, have significantly enhanced the accuracy and accessibility of identifying pathogenic variants in *TSC1* and *TSC2* genes.^26^,^27^

### Limitations:

The limitation of our study lies in its availability of resources, for the present study is based on the detailed data from one family. Future research with larger sample sizes or functional studies to evaluate the effects of variants may provide valuable insights into genotype-phenotype correlations and the clarification of pathogenic mechanisms.

## CONCLUSION

In conclusion, a recurrent heterozygous *TSC2* variant, c.5126C>T (p.P1709L), was identified as the genetic cause of TSC in a Han-Chinese family. The variant leads to an amino acid change from proline to leucine within the functional GAP domain of tuberin. The findings expand the phenotypic spectrum of TSC caused by this variant and may contribute to improve genetic diagnoses and mechanism research of TSC.

### Authors’ contribution:

**XD:** Conceptualization, formal analysis, funding acquisition, investigation, writing—original draft, and writing—review and editing.

**SW:** Conceptualization, formal analysis, visualization, and writing—original draft.

**HD and LY:** Conceptualization, formal analysis, funding acquisition, investigation, resources, writing—original draft, and writing—review and editing.

All authors have approved the final manuscript and are responsible and accountable for the accuracy or integrity of the work.
